# Immunomodulatory effects of nerve stimulator-guided brachial plexus block with laryngeal mask general anesthesia in pediatric upper limb surgery

**DOI:** 10.3389/fped.2026.1753902

**Published:** 2026-04-08

**Authors:** Peng Pang, Shufeng Kang, Qiujun Wang, Longbiao Zhao, Jincheng Wang, Rongtian Kang, Xiaoning Wu, Chunying Zhang, Lijing Hao

**Affiliations:** 1 First Department of Anesthesiology, Hebei Medical University Third Hospital, Shijiazhuang, Hebei, China; 2 Department of Anesthesiology, Hebei Medical University Second Hospital, Shijiazhuang, Hebei, China; 3 Department of Anesthesiology, No. 256 Hospital of Zhengding, Shijiazhuang, Hebei, China

**Keywords:** brachial plexus block, inflammatory factors, nerve stimulator, pediatric upper limb surgery, regulation of immunity, lymphocyte subsets

## Abstract

**Background:**

Pediatric surgical trauma can trigger the body's stress response, leading to immune dysfunction and affecting postoperative recovery. At present, nerve stimulator-guided brachial plexus block has been widely used in children, but its effect on immune function combined with laryngeal mask general anesthesia remains to be clarified.

**Aim:**

This study assessed the impact of combined brachial plexus block and general anesthesia on inflammatory, stress, and immune responses in children undergoing upper limb surgery.

**Methods:**

This retrospective propensity score matching (PSM) cohort study analyzed children undergoing upper limb surgery (June 2022-June 2024). Participants were stratified according to the anesthesia technique used during their upper limb surgery: the observation group received nerve stimulator-guided brachial plexus block, supplemented with laryngeal mask (LMA) general anesthesia, while controls received LMA general anesthesia alone. The primary outcome was the peripheral blood T lymphocyte subsets (CD3^+^, CD4^+^, CD8^+^, CD4^+^/CD8^+^) and inflammatory cytokines (TNF-α, IL-6, IL-10) measured before anesthesia induction (T0), at the end of surgery (T1), and at 6 h (T2), 24 h (T3), and 72 h (T4) postoperatively. Secondary outcomes included stress hormone levels [cortisol [COR], epinephrine [E], norepinephrine [NE]], anesthetic drug dosage, recovery time, extubation time, visual analog scale (VAS) scores, and postoperative adverse reactions.

**Results:**

1:1 PSM yielded 50 matched pairs with balanced baseline characteristics (all *P* > 0.05). The observation group required less intraoperative remifentanil, had shorter recovery and extubation times, and exhibited lower VAS scores at all postoperative time points (all *P* < 0.05). Immunological analyses revealed that the observation group maintained higher CD3^+^ and CD4^+^ cell percentages, along with an elevated CD4^+^/CD8^+^ ratio at T1-T4 timepoints (*P* < 0.05), whereas CD8^+^ cell levels were significantly lower at T2 (*P* < 0.05). Furthermore, the observation group exhibited attenuated inflammatory and stress responses, with lower concentrations of TNF-α, IL-6, cortisol, epinephrine, and norepinephrine at T1-T3, and lower levels of the anti-inflammatory IL-10 at T2 (*P* < 0.05). The overall incidence of postoperative nausea, vomiting, and restlessness was also significantly reduced in the observation group (*P* < 0.05).

**Conclusion:**

Nerve stimulator-assisted brachial plexus block and LMA general anesthesia provides immunoprotection in children, enabling lower drug consumption, faster recovery, and fewer complications, thus proving clinically valuable.

## 1 Introduction

Pediatric upper limb procedures, including fracture management, tendon repair, and nerve reconstruction, are common in pediatric orthopedic and anesthetic practice (1). Compared with adults, children have immature organ function, distinct pharmacokinetics and pharmacodynamics, a stronger stress response to surgical trauma, and lower cooperation (2, 3).

General anesthesia is the mainstay for pediatric upper limb surgery. Laryngeal mask (LMA) general anesthesia is widely used in short-duration pediatric procedures due to its easy airway management, minimal pharyngeal stimulation, and stable hemodynamics during insertion and removal, particularly for upper limb surgery with a limited operative field and duration typically under 2 h (4, 5). However, LMA general anesthesia alone cannot effectively block the transmission of peripheral noxious stimuli to the central nervous system, resulting in a pronounced perioperative stress response (6, 7). Achieving adequate analgesia often necessitates high-dose anesthetics and opioids, consequently increasing risks of delayed recovery, nausea and vomiting, respiratory depression, and agitation (8, 9).

Beyond these clinical drawbacks, accumulating evidence indicates that surgical trauma and general anesthetics themselves can perturb perioperative immune homeostasis (10). Surgery-induced stress activates neuroendocrine pathways and suppresses cell-mediated immunity, characterized by reduced CD3^+^ and CD4^+^ T lymphocyte proportions and a decreased CD4^+^/CD8^+^ ratio, potentially predisposing patients to postoperative infections and delayed recovery (11). Moreover, certain volatile anesthetics and opioids have been shown to exert immunosuppressive effects *in vitro* and in animal models, although their clinical relevance remains debated (12). In children, whose immune system is still maturing, preserving perioperative immune function is particularly critical for preventing complications and promoting rapid recovery (13). Therefore, anesthetic techniques that attenuate surgical stress and minimize exposure to potentially immunosuppressive agents may confer immunoprotective benefits.

To address the clinical limitations of LMA-only anesthesia and the need for immune preservation, an optimized strategy combining regional nerve block with general anesthesia has gained attention. Brachial plexus block provides effective sensory and motor blockade in the upper limb and is the preferred regional technique for upper limb surgery (14–16). Nerve stimulator guidance enables precise localization and improved efficacy while reducing the risk of vascular or nerve injury, which is particularly advantageous in children with immature anatomical landmarks (17, 18). This combined approach integrates the complementary advantages of both techniques: brachial plexus block blunts surgical nociception, attenuating stress response and lowering anesthetic and opioid requirements, while LMA general anesthesia provides satisfactory sedation, ensuring patient comfort and cooperation during surgery (19–21). Studies have found that combined anesthesia can significantly reduce the intraoperative consumption of anesthetic drugs, shorten the postoperative recovery and extubation time, and reduce the incidence of adverse reactions such as restlessness, nausea and vomiting (22–24).

Using a retrospective propensity score matching (PSM) cohort design. While previous investigations have primarily focused on clinical outcomes such as anesthetic consumption and recovery times, the potential immunomodulatory effects of this combined approach remain underexplored. Therefore, the present study placed special emphasis on perioperative immune function and inflammatory responses as primary outcomes. Children were allocated to either combined anesthesia (brachial plexus block supplemented with LMA general anesthesia) or LMA general anesthesia alone. We hypothesized that, compared with LMA general anesthesia alone, nerve stimulator-guided brachial plexus block combined with LMA general anesthesia would: significantly reduce general anesthetic requirements and accelerate early postoperative recovery; provide superior postoperative analgesia (lower VAS scores); attenuate perioperative immunosuppression, inflammatory response and stress response—manifested as higher percentages of CD3^+^ and CD4^+^ cells, a preserved CD4^+^/CD8^+^ ratio, lower levels of pro-inflammatory cytokines (TNF-*α*, IL-6) and stress hormones (COR, E, NE), and a reduced compensatory elevation of anti-inflammatory IL-10; decrease the incidence of postoperative adverse reactions. The results of this study will provide a reliable clinical reference for perioperative anesthesia regimen selection in pediatric upper limb surgery, thereby further optimizing perioperative management and contributing to enhanced patient care and expedited recovery.

## 2 Materials and methods

### 2.1 Study design

**Figure 1:**
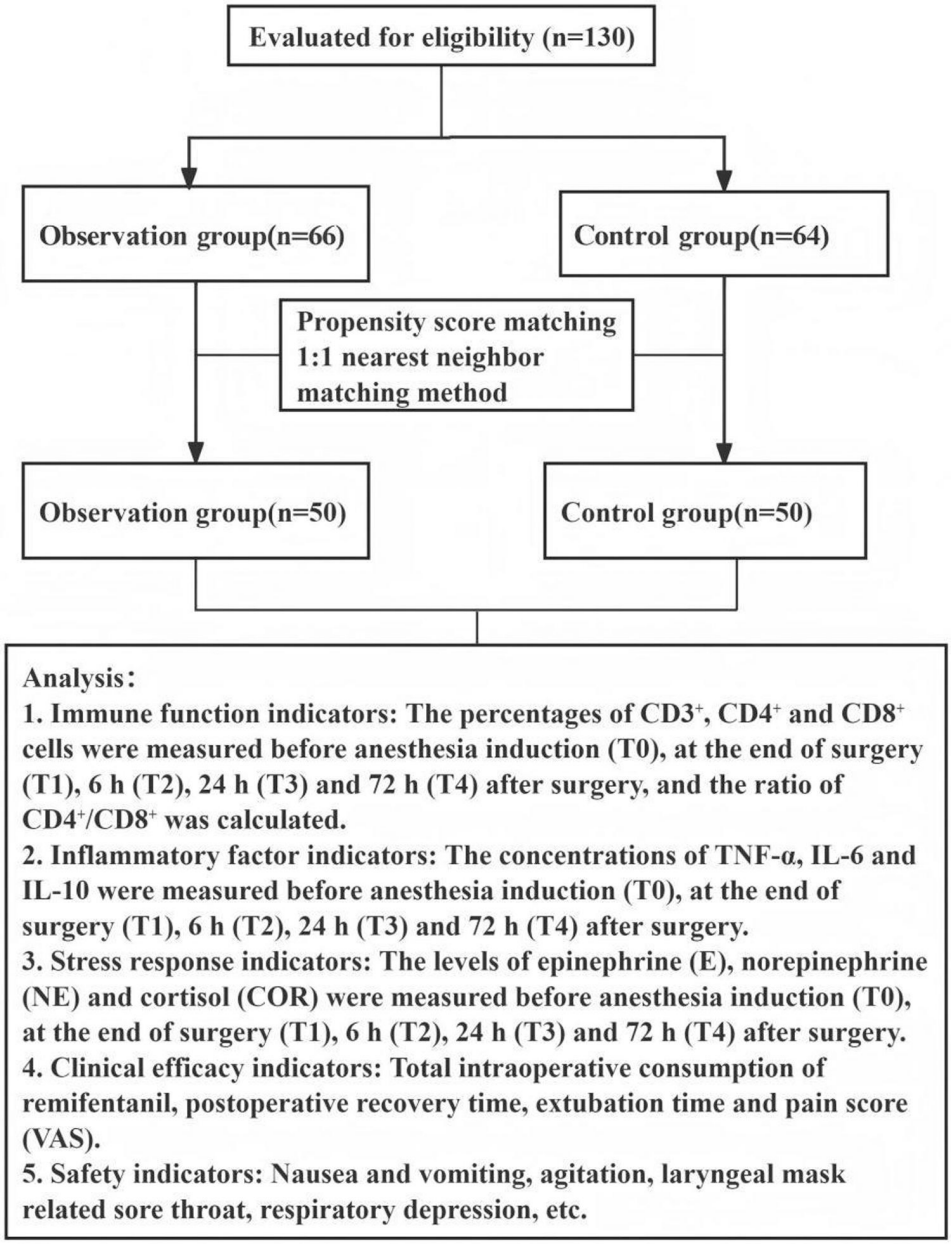
Study flow diagram illustrating the patient selection process, including initial enrollment, propensity score matching (PSM), and final group allocation for children undergoing upper limb surgery with either combined brachial plexus block and LMA general anesthesia (observation group) or LMA general anesthesia alone (control group).

This study was a retrospective PSM cohort study. Children who underwent upper limb surgery in our hospital from June 2022 to June 2024 were enrolled. The anesthetic regimen administered for the surgery was used to categorize patients: those who received brachial plexus blockade combined with LMA general anesthesia formed the observation group, while those who received LMA general anesthesia alone comprised the control group. A schematic diagram of the design is provided in Figure 1.

### 2.2 Ethical explanation

This study received ethics approval from the institutional committee of Third Hospital of Hebei Medical University (approval number: 2024-147-1), complying with the Declaration of Helsinki and international standards for medical research ethics. On account of the retrospective study design, the analysis was based on previous clinical archived data, and the ethics committee has approved the exemption of informed consent. All study data were anonymized to ensure the privacy and identity of the participants.

### 2.3 Inclusion and exclusion criteria

Inclusion criteria: ① with a confirmed diagnosis of upper limb fracture and an indication for surgical management (25); ② Age 3-12 years old; ③ American Society of Anesthesiologists (ASA) grade I or II; ④ type of surgery: unilateral upper limb fracture open reduction and internal fixation, tendon and nerve exploration repair and other non-infectious surgery expected to last within 1-3 h); ⑤ Complete clinical data, all required observation data can be extracted.

Exclusion criteria (26): ① Known to be allergic to local anesthetics (such as ropivacaine) or general anesthetics used in this study; ② Infection, abnormal coagulation function or peripheral neuropathy at the puncture site; ③ Complicated with severe heart, lung, liver, kidney and other important organ dysfunction; ④ patients with autoimmune diseases, recent systemic infection (<4weeks) or long-term use of corticosteroids/immunosuppressants. Patients with recent infection were excluded because pre-existing inflammatory or immune activation would confound the assessment of perioperative immune changes attributable to the anesthetic technique, thereby masking or distorting the true effect of the intervention.; ⑤ Difficult airway was not suitable for LMA ventilation; ⑥ mental or cognitive impairment, unable to cooperate with postoperative evaluation.

### 2.4 Anesthesia methods

The perioperative management of all children in this study followed the established clinical pathway of our hospital. According to the anesthesia records, all children were fasted for 6 h and 2–3 h according to the standard before surgery. Following room entry, venous cannulation was performed while standard parameters (ECG, HR, NIBP, SpO₂, and PetCO_2_) were continuously monitored.

The control group: general anesthesia with LMA (27). Anesthesia was induced by inhalation of 6%–9% sevoflurane at a flow rate of 4–5 L/min. LMA insertion was performed after achieving jaw relaxation, followed by sevoflurane (3%–4%) inhalation at 1 L/min flow rate. Remifentanil was added intermittently during the operation, 10 μg each time. Per anesthesia records, drugs were discontinued 5 min before surgery ended. The LMA was extracted after weaning from oxygen, contingent upon stable hemodynamics, SpO_2_ > 95%, and voluntary eye opening.

The observation group was treated with brachial plexus block guided by nerve stimulator before general anesthesia induction (28). The anesthesia record and surgical nursing record sheet indicated that the intermuscular groove or axillary approach was selected according to the surgical site. The child was placed in the supine position with the head tilted to the contralateral side, the puncture area was exposed and the drape was routinely disinfected. A nerve stimulator (Stimuplex® HNS 12, B/Braun Company, Germany) was used. The initial current was 1. 0 mA and the frequency was 1. After injection of 0.5% ropivacaine 1 mL, the muscle contraction disappeared. At the same time, the diffusion of anesthetic drugs at the injection site can be observed in real time. After confirming that no aspiration occurred, 0.5% ropivacaine 0.5 mL/kg was injected. Following successful sensorimotor blockade confirmation 15–20 min post-procedure, identical general anesthesia protocols were initiated.

### 2.5 Observation indicators

Study data were retrospectively collected from institutional electronic health records, laboratory databases, and nursing documentation systems.

#### 2.5.1 Baseline data

Demographic variables (age/sex/weight/ASA grade) and surgical characteristics (operative time, fracture type), preoperative laboratory indicators (hemoglobin, white blood cell count), and key preoperative inflammatory and stress basal levels (IL-6, TNF-α, COR) were extracted. Relevant data were obtained from medical records, laboratory test reports and nursing records.

#### 2.5.2 Main outcome measures

(1) Immune function indexes: Peripheral blood samples obtained at five perioperative intervals (T0-T4) were analyzed for T-lymphocyte subsets by flow cytometry. Briefly, 2 mL EDTA-anticoagulated whole blood was stained within 2 h with CD3-PerCP-Cy5.5, CD4-FITC, and CD8-PE (BD Biosciences), followed by erythrocyte lysis (FACS™ Lysing Solution). At least 10,000 lymphocyte events were acquired on a FACSCanto™ II cytometer and analyzed using FACSDiva™ software. T-cell subsets were quantified as percentages of CD3^+^ lymphocytes, and the CD4^+^/CD8^+^ ratio was calculated. The gating strategy followed MIFlowCyt guidelines (29); technicians were blinded and consistent reagent lots were used throughout. The percentages of CD3^+^, CD4^+^, CD8^+^ cells and the CD4^+^/CD8^+^ ratio were documented for each time point.
(2) Inflammatory factors: the results of serum samples at the same time point were extracted. According to laboratory records, ELISA measurements determined TNF-α, IL-6, and IL-10 levels in serial serum samples (T0-T4).

#### 2.5.3 Secondary outcome measures

(1) Stress response indicators: the test results of serum samples at the same time point were extracted. At each time point (T0-T4), E and NE levels were detected by enzyme-linked immunosorbent assay, and plasma COR levels were detected by electrochemiluminescence method.
(2) Clinical efficacy indicators: clinical data such as the total amount of remifentanil consumed during operation, postoperative recovery time (from stopping anesthetic drugs to the time when children can open their eyes according to instructions) and extubation time (from stopping anesthetic drugs to successful removal of LMA) were extracted from the anesthesia record sheet.
(3) Postoperative recovery: pain scores at 6 h (T2), 24 h (T3) and 72 h (T4) after operation were extracted from pain nursing records, which were evaluated by VAS, with a score of 0–10 points, with 0 as no pain and 10 as the most severe pain (30, 31).
(4) Safety: the occurrence of adverse reactions such as nausea and vomiting, restlessness, LMA related sore throat and respiratory depression within 72 h after operation were extracted from the course record, nursing record and adverse event report form.

### 2.6 Sample size calculation

G*Power 3.1 software was used to calculate the sample size of this study. IL-10, as a crucial anti-inflammatory cytokinereflecting perioperative immune balance, has been shown to be highly sensitive to anesthesia technique-related modulation in pediatric upper limb surgery, and exhibited the most stable and reproducible between-group differences among multiple immunological endpoints, thereby providing a conservative and reliable effect size estimate. Therefore, this study chose IL-10 as the main effect size parameter for sample size estimation. The t-test formula based on the comparison of the means of two independent samples was calculated, which followed the standard principles of statistical power analysis (32). The main reference was published literature with similar population and type of surgery as this study (27). According to the data of his study, the average value of IL-10 in the control group (general anesthesia only) was predicted to be 27.69 pg/mL on the third day after surgery. In the observation group (combined anesthesia), the predicted average value of IL-10 was 20.37 pg/mL at 3 days after operation. The two-sided significance level (*α*) was 0.05, the power (1 − *β*) was 0.8, and the effect size (*d*) was 0.732. The minimum sample size required for each group was calculated to be 30. To ensure that the final sample size included in the analysis was adequate, and to fully account for the possible sample loss during PSM, as well as the 10% cases of incomplete data or dropout in retrospective studies, the sample size was expanded to 50 cases in each group, and the final total sample size was 100 cases.

### 2.7 Statistical analysis

All analyses used SPSS 26.0, utilizing two-tailed tests at *P* < 0.05 threshold. To address selection bias in this retrospective study, we performed 1:1 propensity score matching with a caliper of 0.02. Matching covariates included age, gender, weight, ASA classification, operative time, fracture location, and preoperative laboratory parameters. Post-matching balance was assessed using standardized differences, with <10% indicating adequate balance. Continuous data normality was verified through Shapiro–Wilk testing. Parametric results appear as mean ± SD (independent t-test), while categorical variables show frequencies (%) (*χ*^2^/Fisher's tests). For repeated measurements of T-cell subsets, inflammatory markers, and stress hormones across timepoints (T0-T4), repeated-measures ANOVA was employed. VAS scores, presented as median (IQR) due to non-normal distribution, were compared using the Mann–Whitney *U*-test. Adverse events are reported as counts (percentages) and analyzed with chi-square or Fisher's exact tests.

## 3 Results

### 3.1 Comparison of baseline data

A total of 130 children were initially enrolled in this study (66 in the observation group and 64 in the control group). In order to control potential confounding bias, we used PSM to perform 1:1 matching, and finally 100 children were successfully matched, with 50 children in each group. The results are shown in Table 1. Before matching, the two groups of children differed in some baseline indicators such as age and weight, Although statistically non-significant (*P* > 0.05), these variations suggested potential selection bias. After PSM, all recorded baseline data, including demographic variables (age/sex/weight/ASA grade) and surgical characteristics (operative time, fracture type), preoperative laboratory parameters (hemoglobin, white blood cell count), and key preoperative inflammatory and stress basal levels (IL-6, TNF-α, COR), were collected. No intergroup differences existed in baseline characteristics or operative duration (*P* > 0.05).

**Table 1:** Baseline characteristics and preoperative laboratory parameters of children before and after propensity score matching.

Indicators	Before PSM				After PSM			
	Observation group (*n* = 66)	Control group (*n* = 64)	statistic	*P*-value	Observation group (*n* = 50)	Control group (*n* = 50)	statistic	*P*-value
Demographics								
Age (years)	6.8 ± 2.3	7.5 ± 2.5	*t* = −1.642	0.103	6.9 ± 2.1	7.0 ± 2.2	*t* = −0.230	0.818
Gender (Male/Female, *n*)	40/26	35/29	*χ²*=0.526	0.468	31/19	29/21	*χ²*=0.160	0.620
Weight (kg)	22.9 ± 5.5	24.8 ± 6.3	*t* = −1.850	0.067	23.4 ± 4.7	23.9 ± 5.4	*t* = −0.498	0.645
ASA Classification (I/II, *n*)	60/6	58/6	*χ²*=0.000	0.989	46/4	47/3	*χ²*=0.000	0.644
Surgical Data								
Operative Time (min)	82.5 ± 19.5	85.3 ± 21.8	*t* = −0.789	0.431	83.1 ± 18.9	84.5 ± 20.4	*t* = −0.355	0.723
Fracture Location (*n*)			*χ*^2^ = 1.058	0.589			*χ*^2^ = 0.784	0.676
Supracondylar Humerus	35 (53.0%)	30 (46.9%)			25 (50.0%)	23 (46.0%)		
Ulna/Radius	19 (28.8%)	18 (28.1%)			15 (30.0%)	14 (28.0%)		
Humerus (Shaft)	12 (18.2%)	16 (25.0%)			10 (20.0%)	13 (26.0%)		
Preoperative Lab Values								
Hemoglobin (g/L)	124.8 ± 9.8	126.1 ± 10.5	*t* = −0.734	0.464	125.2 ± 9.6	124.7 ± 10.0	*t* = 0.258	0.797
White Blood Cell Count (×10⁹/L)	7.6 ± 1.7	7.8 ± 1.9	*t* = −0.649	0.518	7.5 ± 1.6	7.7 ± 1.8	*t* = −0.557	0.579
Preoperative Inflammatory/Stress Markers								
IL-6 (pg/mL)	5.3 ± 1.6	5.1 ± 1.5	*t* = 0.756	0.451	5.2 ± 1.5	5.4 ± 1.6	*t* = −0.614	0.540
TNF-α (pg/mL)	20.8 ± 3.2	20.1 ± 3.0	*t* = 1.303	0.195	20.5 ± 3.1	20.0 ± 3.1	*t* = 0.941	0.349
COR (nmol/L)	212 ± 25	216 ± 27	*t* = −0.882	0.38	215 ± 24	215 ± 24	*t* = 0.012	0.990

PSM, propensity score matching; ASA, American Society of Anesthesiologists; IL-6, interleukin-6; TNF-α, tumor necrosis factor-alpha; COR, cortisol.

### 3.2 Perioperative anesthetic efficiency and clinical outcomes

Perioperative anesthetic and recovery data are summarized in Table 2. Nerve stimulator-guided brachial plexus block was successfully performed in all observation group patients, with a mean performance time of 8.5 ± 2.1 min and a mean local anesthetic volume of 11.7 ± 2.7 mL. Patients receiving combined anesthesia required significantly lower intraoperative sevoflurane concentration and remifentanil consumption compared with the control group (both *P* < 0.001). Consequently, emergence time and extubation time were markedly shortened in the observation group (both *P* < 0.001), indicating faster postoperative recovery.

**Table 2:** Comparison of intraoperative anesthetic drug consumption, emergence time, and extubation time between the two groups.

Indicator	Observation group (*n* = 50)	Control group (*n* = 50)	Statistic	*P*-value
Anesthesia onset time (min)	2.6 ± 1.2	2.7 ± 1.3	*t* = −0.388	0.699
Nerve block performance time (min)	8.5 ± 2.1	–	–	–
Local anesthetic volume (mL)	11.7 ± 2.7	–	–	–
Mean sevoflurane concentration (Vol%)	2.3 ± 0.4	2.9 ± 0.5	*t* = −6.609	<0.001
Remifentanil consumption (µg/kg/min)	0.08 ± 0.02	0.26 ± 0.05	*t* = −23.325	<0.001
Emergence time (min)	6.5 ± 1.8	13.2 ± 2.4	*t* = −15.596	<0.001
Extubation time (min)	8.1 ± 2.0	14.9 ± 2.6	*t* = −14.520	<0.001

min, minutes; mL, milliliter; Vol%, volume percent; µg/kg/min, microgram per kilogram per minute.

### 3.3 Analysis of immune function index results

As presented in Table 3, both cohorts demonstrated comparable baseline T-lymphocyte profiles (CD3^+^, CD4^+^, CD8^+^, CD4^+^/CD8^+^ ratio) at T0 (*P* > 0.05), indicating that the preoperative immune status of the two groups was balanced and comparable. All patients showed a certain degree of cellular immunosuppression during T1 to T3, as indicated by a decrease in the percentages of CD3^+^, CD4^+^ cells and CD4^+^/CD8^+^ ratio. Immunological advantages emerged in the observation group at T1-T3, characterized by higher CD3^+^ and CD4^+^ percentages and a preserved CD4^+^/CD8^+^ ratio; CD8^+^ levels were significantly lower than controls only at T2 (*P* < 0.05), with no intergroup difference at T3. This preserved CD4^+^/CD8^+^ ratios throughout this period (*P* < 0.05). All subsets returned to baseline in both groups by T4 (*P* > 0.05). This indicates that the immune function can eventually be restored under the two anesthesia schemes, but the advantage of the combined anesthesia scheme is that it provides better immune protection during the most critical stress stage (T1 to T3) during the perioperative period.

**Table 3:** Comparison of T-lymphocyte subset percentages (CD3^+^, CD4^+^, CD8^+^, CD4^+^/CD8^+^) at five perioperative time points between the observation and control groups (x ± s, %).

Indicator	Group (*n* = 50)	T0	T1	T2	T3	T4
CD3^+^	Observation group	65.2 ± 4.3	63.1 ± 4.8*	61.5 ± 5.2*	63.8 ± 4.6*	65.0 ± 4.3
	Control group	64.8 ± 4.7	58.3 ± 5.3	55.6 ± 5.8	58.9 ± 5.0	63.5 ± 5.0
CD4^+^	Observation group	38.5 ± 3.8	36.8 ± 3.9*	35.9 ± 4.0*	37.2 ± 3.7*	38.1 ± 3.6
	Control group	38.2 ± 4.0	33.1 ± 4.2	31.2 ± 4.5	33.8 ± 4.1	36.9 ± 3.9
CD8^+^	Observation group	25.1 ± 3.2	24.8 ± 3.3	24.2 ± 3.1*	24.9 ± 3.0	25.3 ± 3.2
	Control group	24.9 ± 3.4	24.5 ± 3.5	26.1 ± 3.6	25.6 ± 3.4	25.0 ± 3.5
CD4^+^/CD8^+^	Observation group	1.53 ± 0.21	1.48 ± 0.20*	1.48 ± 0.22*	1.49 ± 0.19*	1.51 ± 0.20
	Control group	1.54 ± 0.23	1.35 ± 0.24	1.20 ± 0.25	1.32 ± 0.23	1.48 ± 0.22

CD3^+^, CD4^+^, CD8^+^, T lymphocyte subsets; CD4^+^/CD8^+^, ratio of CD4^+^ to CD8^+^ cells; T0, before anesthesia induction; T1, end of surgery; T2, 6 h postoperatively; T3, 24 h postoperatively; T4, 72 h postoperatively.

* *P* < 0.05 compared with control.

### 3.4 Analysis of inflammatory factors

The comparison results of perioperative inflammatory factor levels are detailed in Table 4. Both groups demonstrated comparable, physiologically low concentrations of TNF-α, IL-6, and IL-10 at preoperative baseline (T0) (*P* > 0.05), which excluded the interference of preoperative inflammatory state on the results. All children experienced a peak of inflammatory response from T1 to T2, which showed a sharp increase in the levels of TNF-α, IL-6 and IL-10, among which the increase of IL-6 was the most significant. The observation group demonstrated attenuated inflammatory responses, with significantly lower TNF-α and IL-6 levels at T1-T3 (*P* < 0.05). IL-6 values particularly differed at T2 (68.5 vs. 85.6 pg/mL). Conversely, IL-10 levels were significantly lower in the observation group during this period (*P* < 0.05). By T4, the observation group's inflammatory parameters had normalized, while controls maintained elevated TNF-α. This suggests that the combined anesthetic regimen helps to promote the rapid resolution of the inflammatory state.

**Table 4:** Comparison of serum inflammatory cytokine levels (TNF-α, IL-6, IL-10) at five perioperative time points between the two groups (x ± s).

Indicator	Group (*n* = 50)	T0	T1	T2	T3	T4
TNF-α (pg/mL)	Observation group	20.5 ± 3.1	33.7 ± 4.9*	48.5 ± 6.9*	32.5 ± 4.7*	22.8 ± 3.1
	Control group	19.9 ± 3.1	51.3 ± 6.9	95.8 ± 12.5	75.6 ± 11.4	23.9 ± 3.3
IL-6 (pg/mL)	Observation group	5.2 ± 1.5	45.6 ± 8.8*	68.5 ± 10.3*	52.3 ± 9.2*	13.2 ± 2.9
	Control group	5.4 ± 1.6	65.3 ± 10.6	85.6 ± 11.5	65.6 ± 10.5	14.5 ± 3.9
IL-10 (pg/mL)	Observation group	17.0 ± 2.9	24.2 ± 3.8*	35.2 ± 6.0*	20.4 ± 3.3*	18.1 ± 3.0
	Control group	16.6 ± 2.9	35.5 ± 5.0	48.5 ± 7.1	27.7 ± 4.3	19.3 ± 3.2

TNF-α, tumor necrosis factor-alpha; IL-6, interleukin-6; IL-10, interleukin-10.

* Versus the control cohort at the corresponding time-point

### 3.5 Analysis of stress hormone results

The comparison results of perioperative core stress hormone levels are detailed in Table 5. Preoperative stress hormone levels (COR, E, NE) remained within normal ranges and were comparable between cohorts (T0, *P* > 0.05), which ensured the balance of the starting point of the study. All children experienced a typical stress response during the perioperative period. Stress biomarkers (COR, E, NE) reached maximal concentrations at T2. Throughout T1-T3, the observation group maintained significantly reduced levels vs. controls (*P* < 0.05). All parameters returned to near-baseline at T4 without intergroup differences. This suggests that the combined anesthetic regimen has a major advantage in inhibiting the excessive stress response during the critical period of the perioperative period (from T1 to T3).

**Table 5:** Comparison of stress hormone levels (cortisol, epinephrine, norepinephrine) at five perioperative time points between the two groups (x ± s).

Indicator	Group (*n* = 50)	T0	T1	T2	T3	T4
COR (nmol/L)	Observation group	215 ± 24	224 ± 25*	355 ± 59*	186 ± 21*	220 ± 25
	Control group	215 ± 24	270 ± 31	510 ± 73	231 ± 26	225 ± 25
E (pg/mL)	Observation group	32.5 ± 4.6	53.2 ± 6.9*	55.7 ± 7.9*	40.2 ± 5.5*	35.7 ± 4.6
	Control group	31.9 ± 4.6	81.1 ± 9.7	90.5 ± 14.3	54.4 ± 6.9	37.4 ± 4.7
NE (pg/mL)	Observation group	45.4 ± 5.6	58.6 ± 6.8*	79.4 ± 9.1*	50.8 ± 6.3*	47.0 ± 5.9
	Control group	44.6 ± 5.7	79.4 ± 9.0	108.5 ± 12.4	68.8 ± 7.0	49.0 ± 6.0

COR, cortisol; E, epinephrine; NE, norepinephrine.

* Versus the control cohort at the corresponding time-point.

### 3.6 VAS were compared

The comparison of VAS pain scores between the two groups at each time point after surgery is shown in Table 6. The observation group demonstrated consistently superior analgesia, with significantly lower median VAS scores at all postoperative assessments (T1-T4) (all *P* < 0.001). Specifically, at T1, the median pain score in the control group had reached 3, while the median pain score in the observation group was only 0. At T2, the pain peaked. The median score of the control group was as high as 4 points, indicating moderate to severe pain. With peak VAS scores limited to 1 (indicating mild pain), the observation group maintained significantly better analgesia through T3-T4, confirming the combined technique's superior analgesic efficacy.

**Table 6:** Comparison of postoperative visual analog scale (VAS) pain scores at T1–T4 between the observation and control groups [M (IQR)].

Group	*n*	T1	T2	T3	T4
Observation group	50	0 (0–1)	1 (0–2)	1 (1–2)	1 (0–1)
Control group	50	3 (2–4)	4 (3–5)	3 (2–4)	2 (1–2)
Z-value		−6.812	−7.245	−5.987	−4.512
*P*-value		<0.001	<0.001	<0.001	<0.001

VAS, Visual Analog Scale; M, median; IQR, interquartile range.

### 3.7 Comparison of adverse effects

Adverse event frequencies differed substantially between groups (Table 7). The intervention group experienced reduced postoperative nausea/vomiting (3/50 vs. 11/50, *P* = 0.021) and less emergence agitation (2/50 vs. 10/50, *P* = 0.036). No respiratory depression occurred in either group. LMA-related sore throat incidence was comparably low (*P* = 0.5). The combined technique significantly enhanced pediatric safety by reducing postoperative nausea and vomiting and emergence agitation.

**Table 7:** Comparison of postoperative adverse events between the two groups [*n* (%)].

Group	*n*	Postoperative Nausea and Vomiting	Emergence Agitation	Respiratory Depression	LMA-related Sore Throat
Obsrvation group	50	3 (6.0%)	2 (4.0%)	0 (0.0%)	1 (2.0%)
Contrl group	50	11 (22.0%)	10 (20.0%)	0 (0.0%)	2 (4.0%)
*χ*^2^ value		5.316	4.396	–	–
*P-*value		0.021*	0.036	>0.999	0.5

LMA, laryngeal mask airway.

## 4 Discussion

Children's Physiological function is not mature, and their immune system and stress regulation ability are weak. Traumatic stimulation caused by upper limb surgery can easily cause perioperative stress response and immune function fluctuations, which directly affect postoperative pain control, recovery quality and rehabilitation process. Optimal anesthetic strategy selection is crucial for enhancing pediatric surgical outcomes (33). Through a retrospective propensity score-matched cohort study, this study systematically explored the comprehensive effects of nerve stimulator-guided brachial plexus block combined with LMA general anesthesia on perioperative inflammatory stress response, cellular immune function, and postoperative recovery quality in children undergoing upper limb surgery. The results of our study clearly show that compared with general anesthesia with LMA alone, this combined anesthesia regimen shows significant advantages in many aspects: it not only significantly reduces the dosage of general anesthetics and accelerates postoperative recovery, but also plays a key role in inhibiting excessive inflammatory stress, stabilizing cellular immune function and reducing adverse reactions.

In our study, children in the combination group had a significant reduction in the use of sevoflurane and remifentanil. This phenomenon profoundly reveals the central role of BPB: it changes the focus of anesthesia management from “central potent suppression” relying on high-dose systemic drugs to “peripheral precise intervention” to eliminate noxious stimuli at the source. This shift had multiple benefits. Decreased remifentanil requirements primarily accounted for the observed reduction in postoperative nausea and vomiting. The decrease of total anesthetic drug load directly translates into the shortening of recovery time and extubation time, which lays the foundation for rapid postoperative recovery. In this study, the recovery time and extubation time of the observation group were shortened by nearly 50%, which was consistent with Zhou et al. (34) who confirmed that nerve stimulator-guided brachial plexus block with LMA general anesthesia could shorten the recovery time from 14.1 min to 5.8 min in pediatric upper limb surgery. In addition, Kannan et al. (35) believe that Integrating brachial plexus blockade with general anesthesia enables significant sevoflurane reduction and precise regional analgesia. This strategy enhances pediatric safety by mitigating agitation and promoting rapid recovery.

Stress and inflammatory response triggered by surgical trauma are key pathophysiological processes affecting postoperative outcomes (36). Experimental data revealed simultaneous regulation of stress and inflammatory pathways. The observation group showed significant inhibition of both neuroendocrine activation (COR/E/NE) and pro-inflammatory cytokines, accompanied by lower IL-10 concentrations, suggesting that the combined regimen attenuated the pro-inflammatory response and thus diminished the need for a compensatory anti-inflammatory overshoot. This finding is particularly critical. The sharp increase in IL-10 in the control group is usually a compensatory, even unbalanced, overreaction to a strong proinflammatory response. However, the moderate change of IL-10 in the combined group indicates that the activation level of inflammatory response is low due to the effective blocking of nociceptive afferent signals, so that there is no need to initiate a strong compensatory anti-inflammatory response, and a more balanced and controllable immune internal environment is maintained. This realizes the functional sublation from “analgesia” to “anti-stress and anti-inflammation”. Liu et al. (27) found that postoperative measurements revealed significantly reduced IL-1β (3.47 vs. 4.91 ng/mL) and COR (223.78 vs. 269.54 pg/mL) in the combined vs. general anesthesia group. Wang et al. (37) further verified that Postoperative TNF-α and NE measurements remained persistently low in the intervention cohort 3 days after surgery, which was completely consistent with the trend of inflammatory stress regulation in this study.

Current evidence regarding general anesthetics' immunomodulatory effects remains inconsistent across studies. Relland et al. (38) reported that in adolescents undergoing major spinal fusion surgery, propofol-based total intravenous anesthesia and desflurane inhalation anesthesia resulted in similar degrees of postoperative immunosuppression, suggesting that surgical trauma itself is a dominant driver of perioperative immune dysfunction, potentially overwhelming any subtle differences between anesthetic agents. Our findings are consistent with this perspective.Children receiving combined anesthesia in our study exhibited more stable T-lymphocyte subset profiles (higher CD3^+^, CD4^+^ percentages and CD4^+^/CD8^+^ ratio) compared with those receiving general anesthesia alone. However, because the combined regimen simultaneously reduced both surgical nociceptive input (via brachial plexus block) and the doses of sevoflurane and remifentanil, the relative contribution of each factor cannot be definitively disentangled. Nevertheless, it is biologically plausible that the effective blockade of afferent noxious stimuli plays a predominant role. By attenuating the surgery-induced stress and inflammatory cascades at their origin, regional blockade may preserve cellular immune competence more directly than merely reducing systemic anesthetic exposure. This interpretation aligns with the concept that minimizing surgical trauma-rather than selecting a specific anesthetic agent-is a key strategy for perioperative immunoprotection. From a clinical perspective, optimizing analgesic regimens to reduce the physiological burden of surgery may offer greater benefits than substituting one general anesthetic for another.

Improvements in all biologic measures should ultimately serve to optimize clinical outcomes. In this study, Postoperative VAS evaluations validated the combined anesthesia's analgesic superiority, with consistently reduced pain scores compared to the general anesthesia group. This advantage was verified in multiple dimensions. Zhou et al. (34) found that Emergence agitation rates declined substantially (65.2% to 17.4%) alongside significantly improved VAS scores in the combined anesthesia group. The study by Sengel et al. (39) showed that nerve stimulater-guided block can provide continuous analgesia for up to 9.5 h, completely covering the peak period of acute postoperative pain in children. The combined regimen demonstrated enhanced safety, reducing nausea/vomiting by 73% and agitation by 80% vs. controls. These findings align with Benjamin et al. (40) reporting 65% and 69% reductions respectively in brachial plexus block cohorts. In this study, patients receiving combined anesthesia demonstrated markedly lower agitation rates, which was the inevitable result of the excellent analgesic effect, gentle anesthesia recovery and stable stress state. This outcome indicator profoundly reflects the great value of this combined regimen in improving the perioperative safety and comfort of children.

Beyond statistical significance, the observed improvements in T-lymphocyte subsets and inflammatory cytokines carry meaningful clinical implications. Preservation of CD4^+^ T-helper cells and a higher CD4^+^/CD8^+^ ratio are associated with better cell-mediated immunity, which is critical for defense against opportunistic infections and may reduce the risk of postoperative wound infection or respiratory complications (41, 42). Attenuation of the pro-inflammatory cytokines TNF-*α* and IL-6, along with a blunted compensatory rise of IL-10, reflects a more balanced perioperative inflammatory state. Such immune homeostasis has been linked to faster recovery of organ function, shorter hospital stay, and lower incidence of non-infectious complications such as delirium and acute kidney injury in surgical populations (43, 44). In the pediatric context, where immune reserves are limited and vulnerability to secondary infections is higher, these immunoprotective effects may be particularly valuable. Although our study was not powered to detect differences in rare infectious outcomes, the convergence of improved immunological profiles, enhanced analgesia, faster emergence, and reduced adverse events strongly suggests that the combined anesthetic strategy contributes to a smoother and safer recovery trajectory. Future large-scale, multicenter studies with longer follow-up are warranted to quantify the impact on infection rates, antibiotic utilization, and long-term quality of recovery.

## 5 Conclusion

In Conclusion, this study confirmed the value of nerve stimulator-guided brachial plexus block with LMA general anesthesia for upper limb surgery in children from multiple dimensions such as clinical efficacy, inflammatory stress, cellular immunity and safety. The clinical outcome of enhanced recovery was attained through coordinated effects on anesthetic requirements, inflammatory control, and immunological stability. This protocol not only provides systematic evidence from laboratory indicators to clinical endpoints, but also shows its great potential in optimizing the perioperative management of children. Of course,the study design presents specific limitations. First, though PSM addressed biases inherent to retrospective designs, it could not completely avoid the influence of potential unknown confounding factors. Second, as a single-center study, the extrapolation of the conclusion needs to be verified by more research centers. In addition, our monitoring of immune function ended at 72 h after surgery, which did not reveal more long-term immune recovery. In the future, multi-center trials with extended follow-up should validate long-term recovery benefits.

## Data Availability

The original contributions presented in the study are included in the article/Supplementary Material, further inquiries can be directed to the corresponding author/s.

## References

[1] Naranje SM Erali RA Warner WC Jr. Sawyer JR Kelly DM. Epidemiology of pediatric fractures presenting to emergency departments in the United States. J Pediatr Orthop. (2016) 36:e45–8. 10.1097/bpo.0000000000000595 26177059

[2] Anderson BJ Holford NHG. Tips and traps analyzing pediatric PK data. Paediatr Anaesth. (2011) 21:222–37. 10.1111/j.1460-9592.2011.03536.x 21320233

[3] Habre W Disma N Virag K Becke K Hansen TG Jöhr M Incidence of severe critical events in paediatric anaesthesia (APRICOT): a prospective multicentre observational study in 261 hospitals in Europe. Lancet Respir Med. (2017) 5:412–25. 10.1016/s2213-2600(17)30116-9 28363725

[4] Joshi GP Inagaki Y White PF Taylor-Kennedy L Wat LI Gevirtz C Use of the laryngeal mask airway as an alternative to the tracheal tube during ambulatory anesthesia. Anesth Analg. (1997) 85:573–7. 10.1097/00000539-199709000-00016 9296411

[5] Hartmann B Banzhaf A Junger A Röhrig R Benson M Schürg R Laryngeal mask airway versus endotracheal tube for outpatient surgery: analysis of anesthesia-controlled time. J Clin Anesth. (2004) 16:195–9. 10.1016/j.jclinane.2003.07.008 15217659

[6] Erdoes G Koster A Levy JH. Viscoelastic coagulation testing: use and current limitations in perioperative decision-making. Anesthesiology. (2021) 135:342–9. 10.1097/aln.0000000000003814 33979438

[7] Finnerty CC Mabvuure NT Ali A Kozar RA Herndon DN. The surgically induced stress response. JPEN J Parenter Enteral Nutr. (2013) 37:21s–9. 10.1177/0148607113496117 24009246 PMC3920901

[8] Zeng Y Zhang Y Wu J Li Q Liu F Gao G Optimizing the recovery of pediatric tonsillectomy: application of opioid-free anesthesia and analgesia. J Perianesth Nurs. (2025) 40:1183–90. 10.1016/j.jopan.2024.11.013 40047776

[9] Keles S Kocaturk O. Postoperative discomfort and emergence delirium in children undergoing dental rehabilitation under general anesthesia: comparison of nasal tracheal intubation and laryngeal mask airway. J Pain Res. (2018) 11:103–10. 10.2147/jpr.s153637 29379311 PMC5757964

[10] Stollings LM Jia LJ Tang P Dou H Lu B Xu Y. Immune modulation by volatile anesthetics. Anesthesiology. (2016) 125:399–411. 10.1097/aln.0000000000001195 27286478 PMC5074538

[11] Gaudillière B Fragiadakis GK Bruggner RV Nicolau M Finck R Tingle M Clinical recovery from surgery correlates with single-cell immune signatures. Sci Transl Med. (2014) 6:255ra131. 10.1126/scitranslmed.3009701 PMC4334126 25253674

[12] Yuki K Eckenhoff RG. Mechanisms of the immunological effects of volatile anesthetics: a review. Anesth Analg. (2016) 123:326–35. 10.1213/ane.0000000000001403 27308954 PMC4851113

[13] Sümpelmann R Becke K Zander R Witt L. Perioperative fluid management in children: can we sum it all up now? Curr Opin Anaesthesiol. (2019) 32:384–91. 10.1097/aco.0000000000000727 30925513

[14] Nijs K Hertogen P Buelens S Coppens M Teunkens A Jalil H Axillary brachial Plexus block compared with other regional anesthesia techniques in distal upper limb surgery: a systematic review and meta-analysis. J Clin Med. (2024) 13:3185. 10.3390/jcm13113185 38892896 PMC11173314

[15] Xing T Ge L. Ultrasound-guided brachial Plexus block by costoclavicular space approach: a narrative review. Med Sci Monit. (2023) 29:e939920. 10.12659/msm.939920 37448107 PMC10353486

[16] Guo Z Zhao M Shu H. Ultrasound-guided brachial plexus block at the clavicle level: a review. Drug Discov Ther. (2023) 17:230–7. 10.5582/ddt.2023.01005 37587053

[17] Gurnaney H Ganesh A Cucchiaro G. The relationship between current intensity for nerve stimulation and success of peripheral nerve blocks performed in pediatric patients under general anesthesia. Anesth Analg. (2007) 105:1605–9. 10.1213/01.ane.0000287642.21534.ed 18042857

[18] Singh S Goyal R Upadhyay KK Sethi N Sharma RM Sharma A. An evaluation of brachial plexus block using a nerve stimulator versus ultrasound guidance: a randomized controlled trial. J Anaesthesiol Clin Pharmacol. (2015) 31:370–4. 10.4103/0970-9185.161675 26330718 PMC4541186

[19] Xu C Wang B Yang A Xie Z Liu C Lang X The efficacy of pediatric ultrasound guided brachial plexus block anesthesia and determination of optimal anesthetic drug dosage. Minerva Pediatr (Torino). (2023) 75:171–5. 10.23736/s2724-5276.16.04716-2 27827526

[20] La Palma J Labandeyra H Contreras-Pérez G Luchinni M Sala-Blanch X. Incidence of phrenic nerve palsy in pediatric costoclavicular brachial Plexus block. Paediatr Anaesth. (2025) 35:872–3. 10.1111/pan.70009 40920126

[21] Bae K Kim YJ Lim HW Kang MS Kim HJ Koh WU Evaluating the clinical utility of brachial Plexus block for reducing opioid exposure in pediatric elbow fracture surgery: a retrospective cohort study. Medicina (Kaunas). (2024) 60:483. 10.3390/medicina60030483 38541209 PMC10971820

[22] Miskovic A Johnson M Frost L Fernandez E Pistorio A Disma N. A prospective observational cohort study on the incidence of postoperative sore throat in the pediatric population. Pediatric Anesthesia. (2019) 29:1179–85. 10.1111/pan.13757 31610063

[23] Sharma R Kamal G Agarwal S Gupta A Gupta A Kalra B. Clinical evaluation of two different doses of clonidine as an adjuvant to bupivacaine in ultrasound-guided supraclavicular brachial Plexus block for pediatric upper limb surgeries—a randomized trial. Anesth Essays Res. (2022) 16:244–9. 10.4103/aer.aer_69_22 36447928 PMC9701327

[24] Zadrazil M Opfermann P Marhofer P Westerlund AI Haider T. Brachial plexus block with ultrasound guidance for upper-limb trauma surgery in children: a retrospective cohort study of 565 cases. Br J Anaesth. (2020) 125:104–9. 10.1016/j.bja.2020.03.012 32340734

[25] Bhat TA Gulzar A Bhat AA Bhat TA Ali Z. A review of upper limb injuries in bear maul victims: consistent pattern and inverse relation in severity with facial and scalp injuries. Chin J Traumatol. (2018) 21:38–41. 10.1016/j.cjtee.2017.11.001 29402720 PMC5857895

[26] Liu L Yang F Gao W Li S Tian Y Yang L Median effective volume of 0.2% ropivacaine for ultrasound-guided supraclavicular brachial plexus block in children aged 1-6 years: a prospective dose-finding study. Front Pediatr. (2023b) 11:1157447. 10.3389/fped.2023.1157447 37252041 PMC10213320

[27] Liu YM Li L Zhang FC Ding ZY. Effects of laryngeal mask combined with brachial plexus block on inflammatory responses and stress levels during upper limb fracture surgeries in children. Chongqing Med J. (2022) 51:2374–78+2382. 10.3969/j.issn.1671-8348.2022.14.008

[28] Yang CW Cho CK Kwon HU Roh JY Heo YM Ahn SM. Ultrasound-guided supraclavicular brachial plexus block in pediatric patients -A report of four cases-. Korean J Anesthesiol. (2010) 59(Suppl):S90–4. 10.4097/kjae.2010.59.S.S90 21286471 PMC3030067

[29] Lee JA Spidlen J Boyce K Cai J Crosbie N Dalphin M MIFlowcyt: the minimum information about a flow cytometry experiment. Cytometry A. (2008) 73A:926–30. 10.1002/cyto.a.20623 PMC2773297 18752282

[30] Li L Liu X Herr K. Postoperative pain intensity assessment: a comparison of four scales in Chinese adults. Pain Med. (2007) 8:223–34. 10.1111/j.1526-4637.2007.00296.x 17371409

[31] Beyer JE Mcgrath PJ Berde CB. Discordance between self-report and behavioral pain measures in children aged 3-7 years after surgery. J Pain Symptom Manage. (1990) 5:350–6. 10.1016/0885-3924(90)90029-j 2269802

[32] Faul F Erdfelder E Lang AG Buchner A. G*power 3: a flexible statistical power analysis program for the social, behavioral, and biomedical sciences. Behav Res Methods. (2007) 39:175–91. 10.3758/bf03193146 17695343

[33] Manso MA Guittet C Vandenhende F Granier LA. Efficacy of oral midazolam for minimal and moderate sedation in pediatric patients: a systematic review. Pediatric Anesthesia. (2019) 29:1094–106. 10.1111/pan.13747 31538393 PMC6900062

[34] Zhou H Wang R Cheng R. Application of laryngeal mask anesthesia combined with nerve stimulator-assisted localization for brachial plexus block in pediatric upper limb surgeries. Chin J Pract Med. (2012) 7:88–9. 10.3969/j.issn.1673-7555.2012.36.063

[35] Kannan S Surhonne NS Chethan Kumar R Kavitha B Devika Rani D Raghavendra Rao RS. Effects of bilateral superficial cervical plexus block on sevoflurane consumption during thyroid surgery under entropy-guided general anesthesia: a prospective randomized study. Korean J Anesthesiol. (2018) 71:141–8. 10.4097/kjae.2018.71.2.141 29619787 PMC5903117

[36] Buchman TG Simpson SQ Sciarretta KL Finne KP Sowers N Collier M Sepsis among medicare beneficiaries: 1. The burdens of sepsis, 2012–2018*. Crit Care Med. (2020) 48:276–88. 10.1097/ccm.0000000000004224 32058366 PMC7017943

[37] Wang WK Guo WB Liu H. Study on the application value of laryngeal mask combined with sevoflurane for mild general anesthesia and brachial plexus block in pediatric upper limb fracture surgeries. J Hainan Med Univ. (2018) 24:254–6+261. 10.13210/j.cnki.jhmu.20180105.005

[38] Relland LM Hall M Martin DP Nateri J Hanson-Huber L Beebe A Immune function following Major spinal surgery and general anesthesia. J Pediatr Intensive Care. (2021) 10:248–55. 10.1055/s-0040-1716668 34745697 PMC8561792

[39] Şengel A Büyükfirat E Seçilmiş S Altay N Atlas A Şengül A. Comparison of ultrasound versus ultrasound with nerve stimulator-guided infraclavicular block anesthesia methods in pediatric patients. Medicina (Kaunas). (2025) 61:985. 10.3390/medicina61060985 40572673 PMC12195188

[40] Walker BJ Long JB Sathyamoorthy M Birstler J Wolf C Bosenberg AT Complications in pediatric regional anesthesia: an analysis of more than 100,000 blocks from the pediatric regional anesthesia network. Anesthesiology. (2018) 129:721–32. 10.1097/aln.0000000000002372 30074928

[41] Liu B Li K Li S Zhao R Zhang Q. The association between the CD4/CD8 ratio and surgical site infection risk among HIV-positive adults: insights from a China hospital. Front Immunol. (2023a) 14:1135725. 10.3389/fimmu.2023.1135725 37497209 PMC10366603

[42] Li WJ Peng YX Zhao LQ Wang HY Liu W Bai K T-cell lymphopenia is associated with an increased infecting risk in children after cardiopulmonary bypass. Pediatr Res. (2024) 95:227–32. 10.1038/s41390-023-02765-1 37580551

[43] Chen L. Perioperative inflammation and immune response in anesthesia: implications for recovery and postoperative outcomes. J Cardiothorac Vasc Anesth. (2025) [in press]. 10.1053/j.jvca.2025.11.013 41320622

[44] Saelim K Ruangnapa K Jarutach J Duangpakdee P Surasombatpattana S Prasertsan P. Cytokine profile of post-cardiopulmonary bypass in children. Clin Exp Pediatr. (2025) 68:1015–22. 10.3345/cep.2025.00836 40968612 PMC12672401

